# Co-transforming *bar* and *CsALDH* Genes Enhanced Resistance to Herbicide and Drought and Salt Stress in Transgenic Alfalfa (*Medicago sativa* L.)

**DOI:** 10.3389/fpls.2015.01115

**Published:** 2015-12-16

**Authors:** Zhen Duan, Daiyu Zhang, Jianquan Zhang, Hongyan Di, Fan Wu, Xiaowen Hu, Xuanchen Meng, Kai Luo, Jiyu Zhang, Yanrong Wang

**Affiliations:** State Key Laborotary of Grassland Agro-Ecosystems, College of Pastoral Agriculture Science and Technology, Lanzhou UniversityLanzhou, China

**Keywords:** alfalfa, *bar*, *CsALDH* gene, drought stress, salt stress, transformation

## Abstract

Drought and high salinity are two major abiotic factors that restrict the productivity of alfalfa. By application of the *Agrobacterium*-*mediated* transformation method, an oxidative responsive gene, *CsALDH12A1*, from the desert grass *Cleistogenes songorica* together with the *bar* gene associated with herbicide resistance, were co-transformed into alfalfa (*Medicago sativa* L.). From the all 90 transformants, 16 were positive as screened by spraying 1 mL L^-1^ 10% Basta solution and molecularly diagnosis using PCR. Real-time PCR analysis indicated that drought and salt stress induced high *CsALDH* expression in the leaves of the transgenic plants. The *CsALDH* expression levels under drought (15 d) and salt stress (200 mM NaCl) were 6.11 and 6.87 times higher than in the control plants, respectively. In comparison to the WT plants, no abnormal phenotypes were observed among the transgenic plants, which showed significant enhancement of tolerance to 15 d of drought and 10 d of salinity treatment. Evaluation of the physiological and biochemical indices during drought and salt stress of the transgenic plants revealed relatively lower Na^+^ content and higher K^+^ content in the leaves relative to the WT plants, a reduction of toxic on effects and maintenance of osmotic adjustment. In addition, the transgenic plants could maintain a higher relative water content level, higher shoot biomass, fewer changes in the photosystem, decreased membrane injury, and a lower level of osmotic stress. These results indicate that the co-expression of the introduced bar and *CsALDH* genes enhanced the herbicide, drought and salt tolerance of alfalfa and therefore can potentially be used as a novel genetic resource for the future breeding programs to develop new cultivars.

## Introduction

Drought and high salinity are two major environmental factors that limit the agricultural productivity. These two environmental constraints cause excessive accumulation of aldehydes in plant cells by inducing rapid generation of reactive oxygen species (ROS) and gradually accentuate injury to leaf cell membranes during plant growth through lipid peroxidation (Hou and Bartels, 2014). Plants have evolved complex systems to respond to adverse environmental changes at physiological and biochemical levels, by accumulating compatible solutes and protective proteins, regulating ion absorption and water balance, scavenging reactive oxygen, and more (Tang et al., 2014). However, plants are negatively affected by eliciting rapid and excessive accumulation of ROS, through regulation of the expression of a wide range of stress-responsive genes adapting to various abiotic stress.

Aldehyde dehydrogenases (ALDHs) are NAD (P)^+^-dependent enzymes are considered to be ‘aldehyde scavengeres’ to eliminate toxic aldehydes, due to catalyzing the irreversible oxidation of a wide range of endogenous and exogenous aromatic and aliphatic aldehydes into corresponding carboxylic acids (Zhu et al., 2014). ALDHs are an evolutionary conserved gene superfamily. A series of studies showed that many ALDHs are capable of coping with various abiotic stresses by indirectly reducing lipid peroxidation or detoxifying cellular ROS. Overexpression of *ALDH22A1* in transgenic tobacco plants increased stress tolerance (Huang et al., 2008). Transgenic *Arabidopsis* plants overexpressingn *Ath-ALDH3* showed improved tolerance to osmotic stress, heavy metals, MV, and H_2_O_2_ (Sunkar et al., 2003). Under normal conditions, *ALDH* genes also play vital roles in plant fundamental metabolic pathways. *ALDH* genes contribute to the synthesis and catabolism of a wide of range of biomolecules (Zhu et al., 2014).

Alfalfa (*Medicago sativa* L.) is an important forage legume that is widely grown throughout the world. Alfalfa provides high quality of forage for animals and also improves soil fertility. However, environmental abiotic stress, such as soil salinity and limited water supplies in agriculture are major constraints to the productivity of alfalfa. Moreover, alfalfa yield can be reduced through competition with weeds for growth conditions. Its forage quality can be lowered by decreasing the digestibility and protein content of hay brought about by moderate to severe weed infestation (Cords, 1973; Kapusta and Strieker, 1975). Alfalfa yield from the first herbage cut can be reduced by 60–80% when volunteer monocots are not controlled in the fall. The total alfalfa yield for a season (consisting of three to five herbage cuts) was reduced by 25–35% (Pike and Stritzke, 1984; Ott et al., 1989). Transgenic alfalfa plants grown in a soil medium under glasshouse conditions were phenotypically normal and exhibited bialaphos resistance (Montague et al., 2007). Genetic engineering now has the potential to further improve plant growth and crop productivity by selectively delivering genes that encode for proteins with known enzymatic or structural functions or regulatory proteins involved in stress resistance traits (Newell-McGloughlin, 2014). In our previous study, a *ALDH12A1* gene was cloned from *Cleistogenes songorica*, a xerophytic desert grass, and stress inducible expression of *rd29A*:: *CsALDH* in transgenic *Arabidopsis* plants showed improved tolerance to drought stress (Zhang et al., 2014). This paper reports a study where the *bar* and *CsALDH* genes were co-transformed for the first time into *Medicago sativa* using the *Agrobacterium-mediated* method. The aim of our research was to generate superior alfalfa transformation events with resistance to herbicide and drought or salt stress.

## Materials and Methods

Plant materials sterilized alfalfa (*Medicago sativa* L. cv Jinhuanghou) seeds were germinated on half-strength MS medium in the dark at 24°C for 2 days and at 4°C for 16 h prior to transformation. The *CsALDH12A1* gene from *Cleistogenes songorica* and the *bar* gene driven by CaMV 35S promoter, were introduced into the binary vector pEarlygate101.

### Transformation

The *Agrobacterium* strain GV3101, harboring a binary vector pEarlygate101 *bar*-*35S*::*CsALDH*, was used for the transformation. The T-DNA region of pEarlygate101 *bar*-*35S*::*CsALDH* is illustrated in Supplementary Figure S1.

Overnight cultures of *Agrobacterium* (*OD*_600_ = 0.5) in LB medium, were collected by centrifugation at 5000 rpm for 15 min at 4°C and re-suspended in 30 ml VIM containing MS salt, Gamborg B5 vitamins and 3% sucrose (pH 5.7) to an OD_600_ of 0.5–0.7. The bacterial suspension was maintained in an incubator shaker at 80 rpm, 28°C, for approximately 30 min prior to use.

The transformation and transplanting process was similar to that described by Weeks et al. (2008). The explants were immersed in the *Agrobacterium* suspension. In addition, 0.03% (v/v) silwet77 and 1.8 g sterile white quartz sand were added to excised seedlings and VIM in the 50 mL centrifuge tube. The tube was vortexed for 30 min at the highest speed (setting at ‘8’, 3200 rpm) by vertically placing in a large sample set platform head at room temperature to allow for swirling of the sand in the suspension. Treated seedlings were vertically placed into the co-cultivation medium (0.5 MS medium, 1.5% sucrose, 0.48% agarose, pH 5.8), and supplemented with 2% (v/v) DMSO. Petri plates were sealed with parafilm and incubated in the chamber. Following the co-cultivation period, all seedlings were transferred and vertically placed in SDM, consisting of 1/2 MS medium, 1.5% sucrose, 500 mg/L cefotaxime and 0.48% agarose (pH 5.8). Plates were stored in a growth chamber. The calluses were subcultured every 2 days. After 14 d on SDM, stably transformed and established plantlets were transferred into plastic culture pots (8 cm × 10 cm) containing 80 cm^3^ of vermiculite and perlite (1:1), and they were acclimated to lower humidity in an environmental growth chamber.

### PCR and RT-PCR Authentication

When the plants were at a height of approximately 20 cm, 1 mL L^-1^ 10% Basta solution (8.0 mg l^-1^) was used for preliminary screening. The plants were sprayed three times every 6 days. Genomic DNA was extracted from the alfalfa leaves using a Plant Genomic DNA extraction kit (Tiangen, Beijing). The PCR was conducted with a genomic DNA template in PCR premix using two convergent primers that were complementary to the *CsALDH* gene and another pair of primers that were complementary to the *bar* gene. DNA amplification was performed at 94°C for 3 min; 35 cycles of 94°C for 30 s, 50°C for 45 s, and 72°C for 1 min 30 s, and then a final extension at 72°C for 10 min. Total RNA was extracted using the UNIQ-10 column total RNA extraction kit (Sangon Biotech, Shanghai). The RT-PCR conditions were identical for both *CsALDH* and *MsActin*, as described in the genomic PCR analysis. The PCR products were separated on a 1.2% agarose gel, stained with GelStrain (TransGene Biotech, Beijing), and visualized under UV. Sixteen transgenic plants showing highly expressed *CsALDH* were selected for further stress tolerance and phenotype analysis. All subsequent experiments were conducted on cloned plants from the T_0_ generation of transgenic plants.

### Stress Treatment on Transgenic Plants

For salt stress treatments, plants from the transgenic and WT alfalfa lines were watered every 2 days with 1/8 Hoagland’s nutrient solution for 4 weeks; then, the nutrient solution was supplemented with NaCl. NaCl concentrations were incrementally increased by 50 mM every 2 days until the final concentrations (0, 100, and 200 mM) were achieved. After 10 days of salt treatment, the plants were used for further physiological analysis.

For drought stress treatments, plants from the transgenic and WT alfalfa lines were watered every 2 days with 1/8 Hoagland nutrient solution to field capacity for 4 weeks; after that, the water was withdrawn for15 days (all plants had severe drought stress symptoms). Following this, the soil was rewatered to field water capacity for 7 days. During the drought stress treatments, the physiological indexes were measured at 0, 10, 15 days after moisture stress application and 4 days after rewatering.

### Expression Analysis

Plants were treated with salt stress and drought stress to induce *CsALDH* expression. RNA was used to generate first-strand cDNA with a PrimeScript^TM^ RT reagent Kit and gDNA Eraser (Takara, Japan). Quantitative real-time PCR (q-RT-PCR) was performed on each cDNA template using 2 × SYBR Green PCR Master Mix (Applied Biosystems, USA) on an ABI 7500 real-time PCR system. The transcript levels were calculated relative to the controls and determined using the 2^-ΔΔCT^ method (Zhang et al., 2009). Data represent the means and standard errors of three biological replicates and two technical replicates. The expression levels of the *CsALDH* and *MsActin* were analyzed by q-RT-PCR using the gene-specific primers listed in Supplementary Table S1.

### Phenotyping and Physiological Analysis of Transgenic Plants

Plant height was determined by measuring the stem length from the top of the shoot apex to the base of the stem under natural growth conditions. The shoot biomasses of all plants were rapidly clipped and weighed fresh.

The relative water content (RWC) levels were measured using the procedures described by Ahmad et al. (2008). Soil water content (SWC): the soil samples were dried at 105°C until they had a constant mass; then, the percentage of soil loss and dry soil mass was used to determine the SWC.

The chlorophyll content was measured with a portable chlorophyll meter (SPAD-502, Konica Minolta, Japan) on the intact fully expanded fifth leaf (from the top) of individual plants. Before measurement of the maximum quantum yield of photosystem II photochemistry (*Fv*/*Fm*), all plants were maintained in the dark for 30 min followed by the *Fv*/*Fm* measurement using a portable modulated chlorophyll fluorometer (PAM-2100, Germany). The measurement was conducted on intact fully expanded leaves of individual plants. The net photosynthetic rate (Pn) was measured using an automatic photosynthetic measuring apparatus (LI-6400, USA), as described by Qiu et al. (2003).

The malondialdehyde (MDA) content was determined according to a modified thiobarbituric acid (TBA) method (Kim and Nam, 2013). The free proline content was spectrophotometrically measured according to the method of Bates et al. (1973).

The Na^+^ and K^+^ levels were measured according to the method described by Flowers and Hajibagheri (2001) with slight modifications. The leaves from the transgenic and WT plants of each treatment were dried at 80°C for 48 h, followed by dry weight measurement. Na^+^ and K^+^ were extracted from dried plant tissue with 100 mM acetic acid at 90°C for at least 2 h. The cation contents was then determined using a flame spectrophotometer (2655–00, ColeParmer Instrument Co., USA) (Bao et al., 2009).

### Statistical Analyses

All assays were biologically replicated at least three times. The data were evaluated with Statistical Package for the Social Sciences (SPSS 16) and Excel. The means were separated using Duncan’s multiple range test at *p* = 0.05.

## Results

### Regeneration of Transgenic Plants

pEarlygate101 *bar*-*35S*::*CsALDH* was transformed into *Medicago sativa* with the *Agrobacterium-mediated* method. In total, 90 T0 transgenic alfalfa plants after antibiotics selection were transplanted into pots for further analyses.

Transgenic plants were preliminarily screened by spraying 1 mL L^-1^ 10% Basta solution. After 18 days of observing the growing conditions, although most of the regenerated plants thrived, some transplanted, regenerated plants still had yellow coloration or had died; these plants may have not contained the herbicide resistance *bar* gene. Under the same conditions, the WT alfalfa died. After spraying Basta solution, 54 transgenic plants with the *CsALDH* gene were obtained and evaluated by further molecular authentication (Supplementary Figure S2A).

### PCR, RT-PCR, and q-RT-PCR Analysis

**FIGURE 1:**
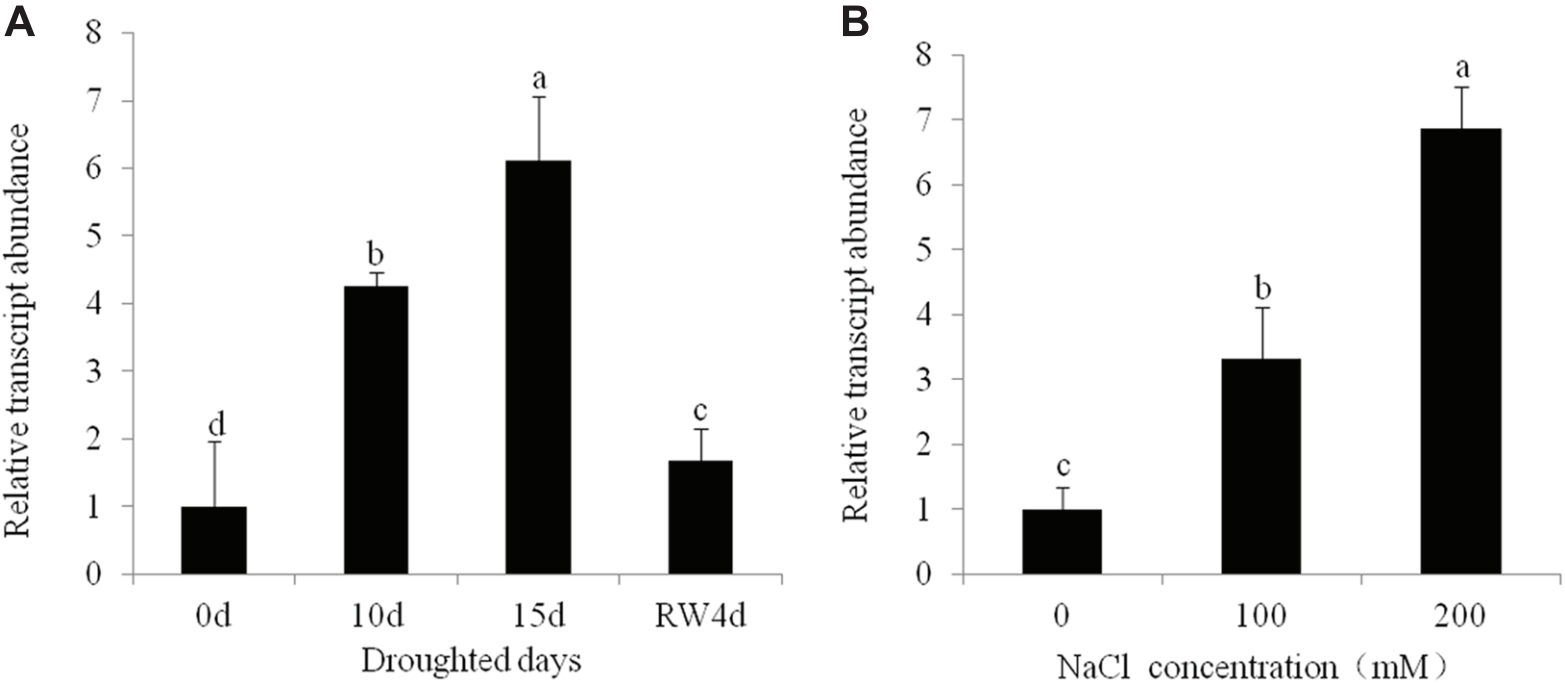
**Expression pattern of *CsALDH* gene in transgenic alfalfa under drought and salt stress. (A)** Drought stress at 0, 10, 15 days after withdrawn watering and 4 days after rewatering; **(B)** salt stress with NaCl concentrations at 0, 100, and 200 mM. *P* < 0.05.

Putative T_0_ transgenic alfalfa plants was initially screened by genomic PCR and RT-PCR analyses. Of the 54 plants that survived, 16 displayed the diagnostic bands for the *CsALDH* gene (about 1400 bp) (Supplementary Figure S2B) and *bar* gene (about 450 bp) (Supplementary Figure S2C) by PCR. The PCR positive rate was 29.6%. The results of RT-PCR analysis indicated that *CsALDH* was effectively expressed in all PCR positive plants (Supplementary Figure S2D). The expression patterns of *CsALDH* during drought and salt stress were evaluated using q-RT-PCR analysis. The drought and salt stress induced dramatically the increased gene expression in leaves (*P* < 0.05). The *CsALDH* expression levels in leaves with 15 d of drought and 10 d of 200 mM NaCl treatment are 6.11 times and 6.87 times, respectively, higher than the control levels (**Figure 1**).

### Overexpression of *CsALDH* Increased Drought Tolerance in Transgenic Alfalfa

**FIGURE 2:**
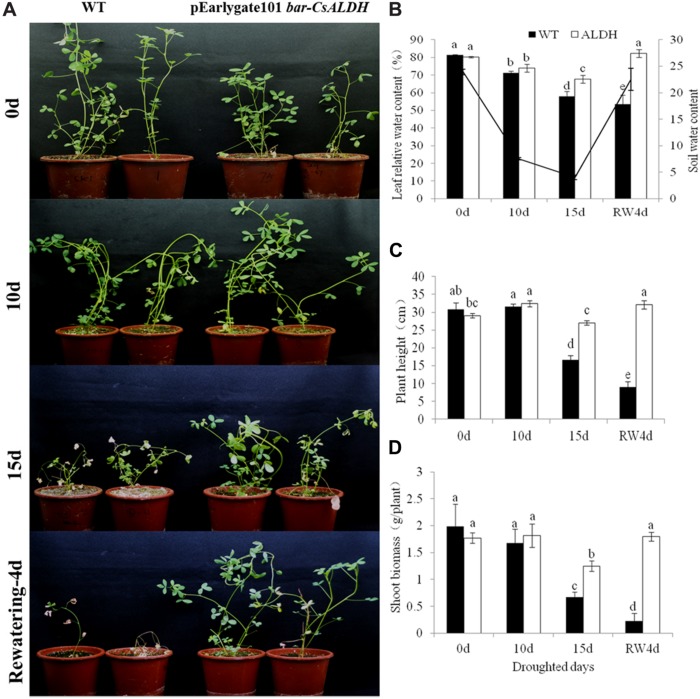
**Drought stress tolerance of *CsALDH* transgenic plants. (A)** Drought stress at 0, 10, 15 days after withdrawn watering and 4 days after rewatering; **(B)** leaf relative water content (RWC) and soil water content (SWC) of WT and transgenic plants; **(C)** plant height of WT and transgenic plants; and **(D)** shoot biomass of WT and transgenic plants. WT, wild-type plants; *CsALDH*, transgenic alfalfa co-expressing bar and *CsALDH. P* < 0.05.

The phenotype observation indicated that after 15 days of drought stress, the leaves of WT alfalfa turned yellow, while the transgenic alfalfa only wilted. After rewatering, the transgenic plants recovered to the normal phenotype, while the wild-type plants could not be restored (**Figure 2**).

Plant height and shoot biomass were also measured in the different drought stress treatments. Plant height and shoot biomass of the WT plants decreased gradually with increased days in drought. While these two traits in the transgenic plants had little difference. In the first 10 days, the transgenic plants grew normally, plant height and shoot biomass increased slightly. There was a slight decline under the 15 days of drought stress conditions. The transgenic plants resumed normal growth after 4 days of rewatering. These observations suggested that the overexpression of *CsALDH* confers the transformed alfalfa with an increased drought tolerance.

To study the osmoregulatory capacity in alfalfa, the RWC levels were evaluated during water deprivation. The RWC levels were reduced in both transgenic and WT plants, but the RWC level in WT plants had a faster decline than in transgenic alfalfa according to time-lapse analysis. This observation suggested that overexpression of *CsALDH* enhanced the capacity for osmotic adjustment of transgenic alfalfa, resulting in greater water uptake at low SWC during drought stress.

**FIGURE 3:**
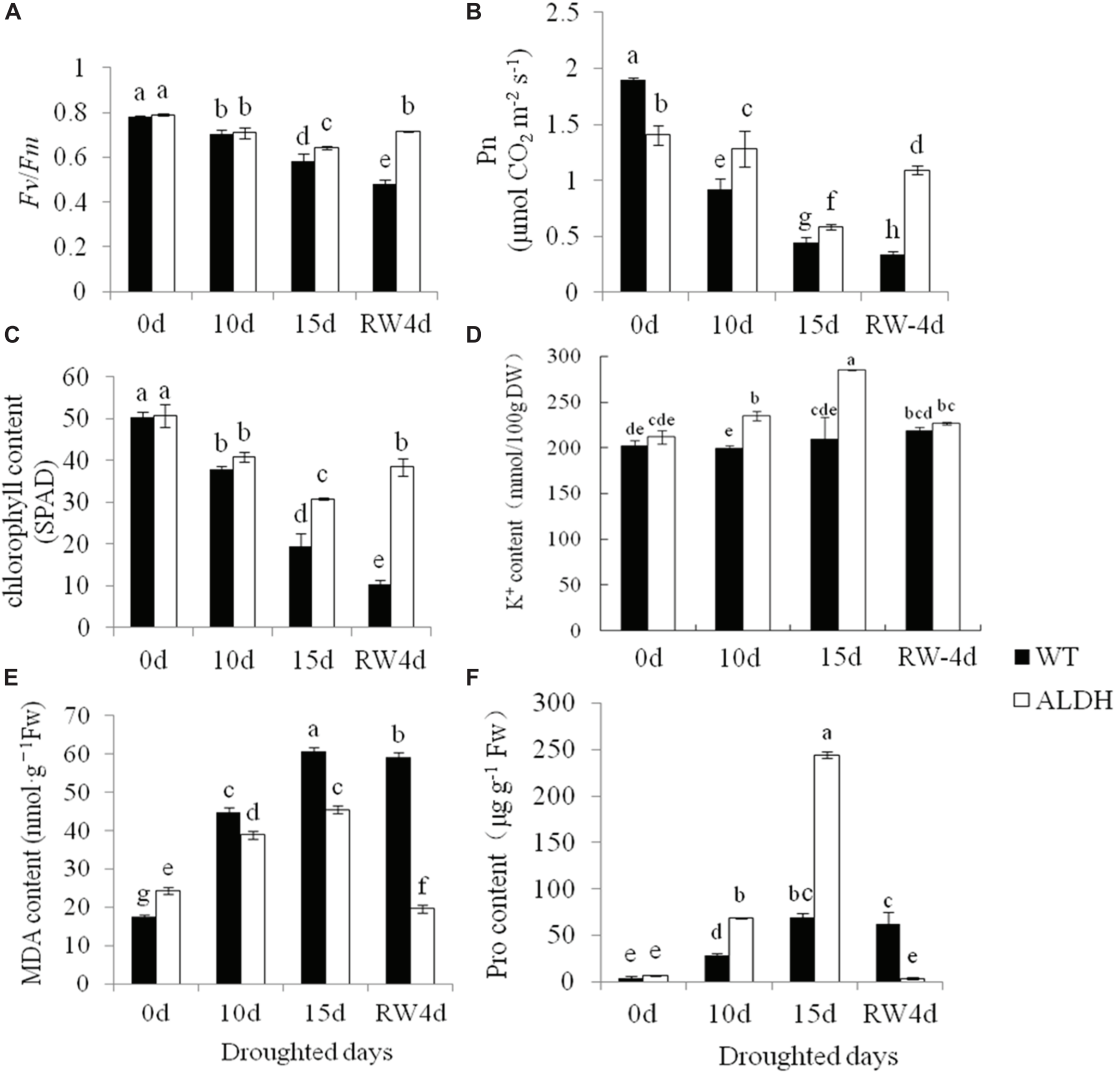
**Measurement of physiological changes under drought-stress treatment**. Drought stress at 0, 10, 15 days after withdrawn watering and 4 days after rewatering. **(A)** Chlorophyll fluorescence (*Fv*/*Fm*); **(B)** net photosynthetic rate (Pn); **(C)** chlorophyll content; **(D)** K^+^ content; **(E)** MDA content; and **(F)** proline content. *P* < 0.05.

To further evaluate the increased drought tolerance of transgenic alfalfa overexpressing *CsALDH*, the chlorophyll content, chlorophyll fluorescence (*Fv*/*Fm*) and net photosynthetic rate of the WT and transgenic plants were determined (**Figures 3A–C**). Chlorophyll fluorescence was monitored as a reflection of photosystem II activity. Before drought stress, there was no significant (*P* > 0.05) difference in the ratio of *Fv* to *Fm* (*Fv*/*Fm*) in the leaves of WT and transgenic plants. However, after the drought treatment, the transgenic plants maintained high levels of *Fv*/*Fm* compared to WT plants. In addition, although the net photosynthetic rate and chlorophyll content decreased in all experimental plants with an increase in the drought time, the Pn and chlorophyll levels of the WT plants had a greater decrease compared to transgenic alfalfa. After rewatering, the Pn and chlorophyll content of the transgenic alfalfa returned to the pre-drought stress levels, while the WT plants still exhibited a decline, indicating that the photosystem of transgenic plants was less affected than that of the WT plants during the water-deficit treatment. The photosystem of the WT plants may have been destroyed under the extreme drought conditions.

The MDA content represents the degree of cell membrane damage. Results showed that the MDA content of WT and transgenic plants increased after the water-deficit stress, but the MDA content of WT plants was higher than that of transgenic plants, indicating that the degree of membrane injury in WT plants would be higher than that of transgenic plants (**Figure 3E**). When suffering from abiotic stress, plants can accumulate more compatible osmolytes, such as free proline, that function as osmoprotectants so that plants can tolerate stress. Therefore, the proline content was tested in transgenic and WT plants (**Figure 3F**). Under the control conditions, the content of free proline was not significantly (*P* > 0.05) different between the WT and transgenic plants. However, after drought treatment, the transgenic plants accumulated higher levels of free proline than the WT plants. In addition to the accumulation of free proline and other soluble organic osmoticum to osmoregulation, alfalfa also adapted to drought stress by absorbing K^+^ to osmotic adjustment. In this study, the contents K^+^ were determined in the leaves of transgenic plants and WT plants at different water-deficit treatments (0, 10, and 15 days, and then 4 days of rewatering) (**Figure 3D**). The transgenic plants accumulated more K^+^ in their leaves, but no significant (*P* > 0.05) increase could be observed in the WT plants.

### Overexpression of *CsALDH* Increased Salt Tolerance in Transgenic Alfalfa

**FIGURE 4:**
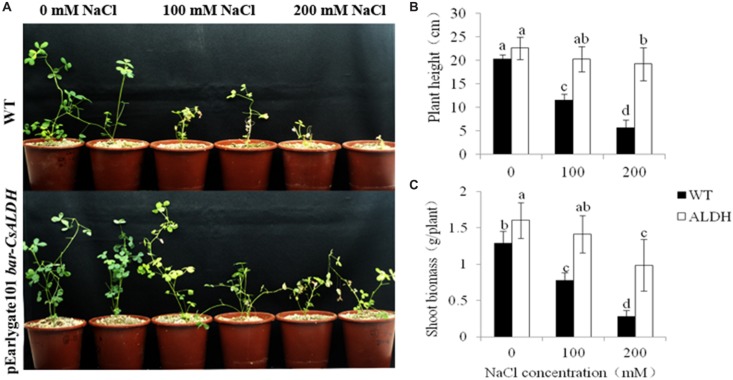
**Salt stress tolerance of *CsALDH* transgenic plants**. salt stress with NaCl concentrations at 0, 100, and 200 mM. **(A)** Growth of WT and *CsALDH* transgenic plants under salt stress; **(B)** plant height of WT and transgenic plants; and **(C)** shoot biomass of WT and transgenic plants. *P* < 0.05.

To assess whether *CsALDH* is also associated with salt stress tolerance, the transgenic and WT plants were irrigated with 0, 100, and 200 mM NaCl solution for 10 days. Phenotypically, the transgenic plants did not differ from the WT plants under normal control growth conditions. After watering with salt solutions (100 and 200 mM of NaCl) for 10 days, the WT plants showed chlorosis and their growth ceased under the treatments over 100 mM NaCl. However, transgenic alfalfa plants were only affected slightly; they stayed green and continued to grow. At 200 mM NaCl, the WT plants almost died while the transgenic plants exhibited chlorosis (**Figure 4A**).

To further study the effect of salinity on plant height and shoot biomass expression in the transgenic and WT alfalfa plants, these traits were measured from the different treatments of NaCl concentrations. The plant height and shoot biomass of all plants decreased gradually with increased salt concentrations. However, expression of these two traits in the WT plants were significantly (*P* < 0.05) lower than transgenic plants at the same NaCl concentration (**Figures 4B,C**).

**FIGURE 5:**
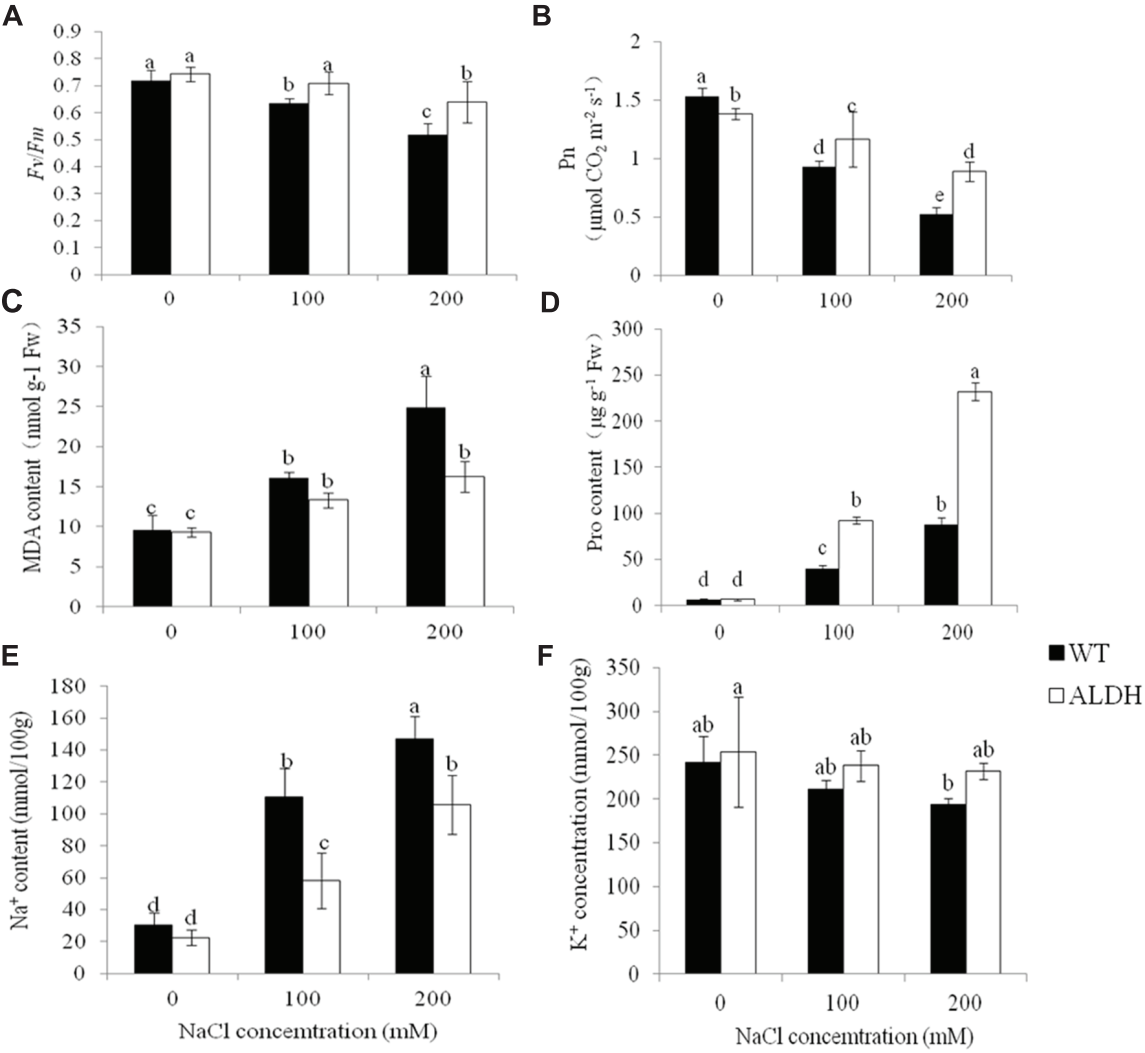
**Measurement of physiological changes under salt-stress treatment**. salt stress with NaCl concentrations at 0, 100, and 200 mM. **(A)** Chlorophyll fluorescence (*Fv*/*Fm*); **(B)** net photosynthetic rate (Pn); **(C)** MDA content; **(D)** proline content; **(E)** Na^+^ content; and **(F)** K^+^ content. (*P* < 0.05).

The chlorophyll content, chlorophyll fluorescence *(Fv*/*Fm*) and net photosynthetic rates of the WT and transgenic plants were also determined to evaluate the increased salt tolerance of transgenic alfalfa overexpressing *CsALDH* (**Figures 5A,B**). Before salt stress, there was no significant difference (*P* > 0.05) in the *Fv*/*Fm* or chlorophyll content in the leaves of the WT and transgenic plants; only the net photosynthetic rate showed an exceptional phenomenon in which the Pn in transgenic plants was lower than that in WT, which may indicate that it is dormant. However, after the NaCl treatment, transgenic plants maintained high levels of *Fv*/*Fm*, chlorophyll content and Pn compared to the WT plants. Additionally, although the *Fv*/*Fm*, chlorophyll content and Pn decreased in all experimental plants with the increase in NaCl, the *Fv*/*Fm*, chlorophyll level and Pn of WT plants decreased more than the levels detected in the transgenic alfalfa.

The MDA content was also examined (**Figure 5C**). Under control conditions, the MDA content of transgenic plants was comparable to that of WT plants. After 10 days of treatment with different NaCl concentration treatment (0, 100, and 200 mM), the MDA content of WT and transgenic plants increased. The MDA content of transgenic plants was lower than that of WT plants. This indicated the lower membrane injury of transgenic plants. To determine whether *CsALDH* overexpression impacted on the proline content, the proline content in the transgenic and WT plants was measured (**Figure 5D**). No significant differences in the proline content were detected between the WT and transgenic plants at 0 mM NaCl. Significantly higher proline was detected when the transgenic plants were treated with 100 and 200 mM NaCl than WT plants, indicating that proline was involved in salt tolerance (*P* < 0.05).

To ascertain whether *CsALDH* gene expression altered the Na^+^ and K^+^ absorption under salt stress conditions, the accumulation of Na^+^ and K^+^ in the NaCl-treated transgenic and WT alfalfa plants was measured (**Figures 5E,F**). The NaCl treatment significantly increased the cellular Na content in both the transgenic and the WT plants (*P* < 0.05). However, at 100 and 200 mM NaCl, there was significantly more Na^+^ in the transgenic plants in comparison to the WT plants. Although the K^+^ levels decreased with increasing NaCl concentrations in the leaves of both the transgenic and WT plants, the K^+^ content in the transgenic plants was significantly (*P* < 0.05) higher than that of the WT plants.

## Discussion

The ALDHs superfamily consists of diverse enzymes involved in endogenous and exogenous aldehyde metabolism. Active ALDHs have been demonstrated to be required in the detoxification of aldehydes by oxidation to their corresponding carboxylic acids and a large number of ALDHs have been shown to be involved in improvement of stress tolerance (Rodrigues et al., 2006; Huang et al., 2008; He et al., 2014; Zhang et al., 2014). In this study, *CsALDH* was overexpressed in alfalfa together with the *bar* gene, which provides herbicide resistance. The results from this study indicated that overexpression of *CsALDH* significantly (*P* < 0.05) enhanced the drought and salt tolerance of transgenic alfalfa plants.

Previous studies have reported that the maintenance of high cytosolic K^+^/Na^+^ ratios could be crucial for salt-tolerant plants (Ren et al., 2005). The transgenic alfalfa plants, co-expression of *ZxNHX* and *ZxVP1-1*, resulted with more Na^+^, K^+^, and Ca^2+^ accumulation in leaves and roots (Bao et al., 2015). By analyzing the toxic effects of ions, we found that transgenic alfalfa overexpressing *CsALDH* had decreased Na^+^ and increased K^+^ levels, resulting in a higher K^+^/Na^+^ ratio compared to the WT plants (**Figures 5E,F**). These results indicated that *CsALDH* positively regulates the salt tolerance of alfalfa by regulating the K^+^/Na^+^ homeostasis to reduce ion toxicity.

Abiotic stress results in the excessive accumulation of ROS in plants, leading to lipid peroxidation. Because MDA is an end-product of lipid peroxidation in biomembranes, the MDA content reflects the extent of lipid peroxidation and membrane injury. Oxidative stress-induced membrane damage has been used as efficient criteria for assessing the degree of salt and drought tolerance of plants (Sairam et al., 2005; Zhang et al., 2014). Overexpressing *ScALDH21* tobacco plants showed higher growth ratio, higher proline accumulation, lower MDA contents, and stronger photosynthetic capacities, when subjected to drought and salt stress (Yang et al., 2015). We found that the MDA content of the transgenic alfalfa was lower under both salt and drought stress compared to the WT plants. These results imply that the degree of membrane injury of transgenic plants was less than that of WT plants, which is in agreement with the enhanced salt tolerance phenotype of transgenic alfalfa.

Osmotic adjustments, by accumulating osmoprotectants inside the cell, are essential for reducing the cellular osmotic potential against an osmotic gradient between root cells and the outside saline solution, which eventually restores the water uptake into roots during salinity stress (Li et al., 2014). Proline and soluble sugar often osmoprotectants, allow the plants to tolerate stress. In this study, we examined the contents of the osmoprotectant proline in plants. The free proline levels were higher during drought and salt stress conditions, and the free proline levels in *CsALDH*-overexpressing transgenic plants were higher than those in WT plants (**Figures 3F** and **5D**). Therefore, the increased accumulation of proline contributes to the increased drought and salt tolerance of transgenic alfalfa. Intriguingly, analysis of the K^+^ level of the plants that were subjected to different levels of drought stress revealed that the transgenic plants accumulate more K^+^ in their leaves, but no significant increase could be observed in the WT plants. This could indicate that the plants absorb K^+^ as part of osmotic adjustment when adapting to drought stress.

The lack of water and salinity limit photosynthetic capacity. In fact, the *Fv*/*Fm* ratio, a parameter commonly known as the maximum quantum yield of primary photochemistry or the photochemical efficiency of PS II (Daud et al., 2015), is used as a basic tool in plant photosynthetic research for stress studies. The total chlorophyll content and Pn decreased under saline conditions. Gama et al. (2007) reported that a reduction in plant growth is associated with a reduction in photosynthesis. Percival et al. (2003) mentioned that the leaf chlorophyll fluorescence responses to increasing salinity were reduced. The reduction in *Fv*/*Fm* due to salinity stress could be related to chlorophyll damage under saline conditions (Ganieva et al., 1998). Our results showed that the *Fv*/*Fm* ratio and Pn decreased as the degree of drought and salt stress increased, and their levels in *CsALDH*-overexpressing transgenic plants were higher than those in WT plants during drought and salt stress. As a result, the increased *Fv*/*Fm*, chlorophyll content and Pn likely contributed to the increased stress tolerance of transgenic alfalfa.

## Conclusion

The exogenous *CsALDH* gene and *bar* gene were co-transformed into the most important forage legume crop, alfalfa, with the *Agrobacterium*-*mediated* transformation method. The transgenic plants grew better than the WT during drought and salt stress conditions and, at worst, wilted slightly, whereas the growth of WT plants was stunted. Many WT plants did not survive after rewatering, while the transgenic plants resumed normal growth. The overexpression of the *CsALDH* gene in the alfalfa genome enhanced its drought and salt tolerance through improving plant growth, RWC, membrane protection, compatible osmolytes, chlorophyll fluorescence, chlorophyll contents, and Pn. The *CsALDH* and *bar* transgenic alfalfa plants generated as resistant sources in this study had improved stress and herbicide tolerance, which could help in weed control resulting in production costs and enhancing the growth of the alfalfa cultivars during drought and in salty soils. However, field experiment need to be arranged for further evaluation of stress and herbicide tolerance in *CsALDH* and *bar* transgenic alfalfa plants.

## Author Contributions

Conceived and designed the experiments: JZ, YW, XH, ZD. Performed the experiments: ZD, JZ, DZ. Ana-lyzed the data: ZD. Contributed reagents/materials/analysis tools: ZD, KL, DZ, HD, FW, XM. Wrote the paper: ZD.

## Conflict of Interest Statement

The authors declare that the research was conducted in the absence of any commercial or financial relationships that could be construed as a potential conflict of interest.
