# Supplementary material for: Co-transforming bar and CsALDH Genes Enhanced Resistance to Herbicide and Drought and Salt Stress in Transgenic Alfalfa (Medicago sativa L.)
Source: Front Plant Sci. 2015 Dec 16;6:1115. doi: 10.3389/fpls.2015.01115 (PMC4679862; doi:10.3389/fpls.2015.01115)
Supplement: Supplementary file 1 [file Data_Sheet_1.DOC]

**Supplement Tables and Figures Legend**

Supplement Table 1. Primers used for PCR assays

Supplement Fig. 1. Map of the T-DNA region of plasmid pEarlygate 101 *bar-ALDH*

Supplement Fig. 2 Verification of transgenic plants

Supplement Table 1. Primers used for PCR assays

| Primer name | (5'-3') Nucleotide sequence | Purpose | |
| --- | --- | --- | --- |
| *CsALDH*_F | AATTGGTCATCTAGCGGGCTTCT | PCR detection for *CsALDH* gene | |
| *CsALDH*_R | TCATCCTTTCACAGGCTTCC |  |  |
| *Bar*_F | GCAGGAACCGCAGGAGTGGA | PCR detection for *bar* gene | |
| *Bar*_R | ATCTCGGTGACGGGCAGGAC |  | |
| *CsALDH*_F_Qrt_ | CATTCCGGGTGGTGACTGAAT | Q-RT-PCR of *CsALDH* | |
| *CsALDH*_R_Qrt_ | GTACCGTTGACCGATCTCCC |  | |
| *MsActin*-F | GAGACTTTCAATGTGCCCG | Q-RT-PCR of *MsActin* | |
| *MsActin*-R | AGCATGTGGGAGTGCATAAC |  | |


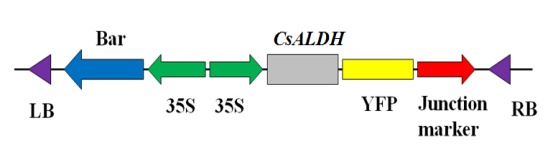


Supplement Fig. 1 Map of the T-DNA region of plasmid pEarlygate 101 *bar-CsALDH.* *CsALDH* cloned from *Cleistogenes songorica*, and *bar* gene, motivated by *CaMV 35S* promoter, were constructed into pEarlygate 101 vector by gateway method.


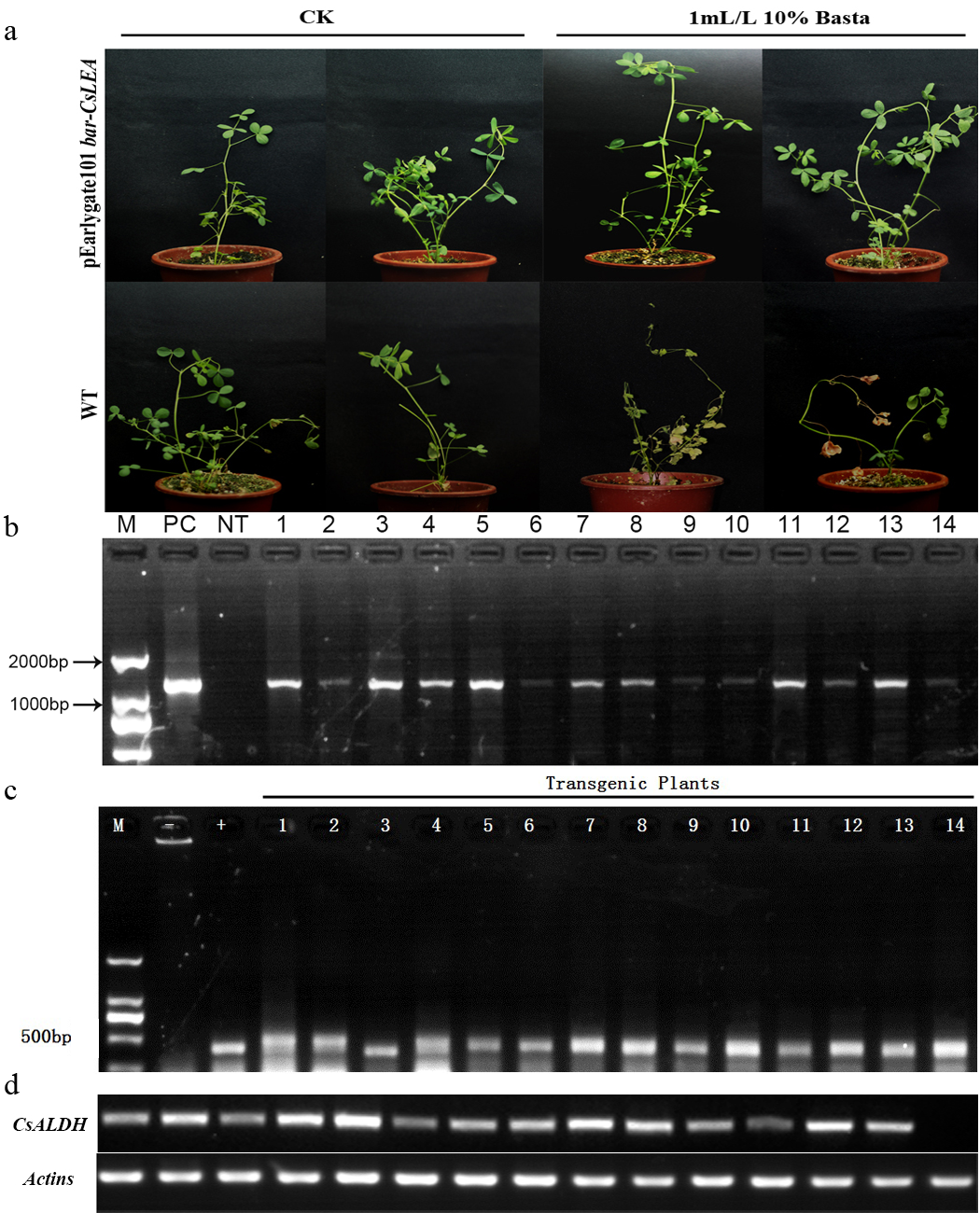


Supplement Fig. 2 Verification of transgenic plants

a. Basta stress tolerance of transgenic alfalfa. left: Plants growth under normal conditions. right: Plants growth under herbicide stress, 1 mL L^-1^ 10 % Basta solution (8.0 mg l^−1^) was sprayed three times every 6 d. b. Amplification of *ALDH* gene from the survived transgenic plants, with an expected amplification size of about 1400bp for *CsALDH* gene. c. Amplification of *bar* gene from the survived transgenic plants, with an expected amplification size of about 450 bp for *bar* gene. M. DL2000 DNA Marker; PC, positive control; Numbers (1-17) represent independent transgenic lines. d. Amplification of *ALDH* gene by RT-PCR, amplification size of about 412 bp.
